# Effects of visual scanning exercises in addition to task specific approach on balance and activities of daily livings in post stroke patients with eye movement disorders: a randomized controlled trial

**DOI:** 10.1186/s12883-022-02843-7

**Published:** 2022-08-24

**Authors:** Sana Batool, Hamayun Zafar, Syed Amir Gilani, Ashfaq Ahmad, Asif Hanif

**Affiliations:** 1 Faculty of Allied Health Sciences, Department University Institute of Physical Therapy, The University of Lahore, Lahore, Pakistan; 2 Department of Rehabilitation Sciences, College of Applied Medical Sciences, King Saud University, Riyadh, Saudi Arabia; 3 Rehabilitation Research Chair, King Saud University, Riyadh, Saudi Arabia; 4 Department of Odontology, Clinical Oral Physiology, Faculty of Medicine, Umea University, Umea, Sweden; 5 Faculty of Allied Health Sciences, Directorate of International Linkages, University of Lahore, Lahore, Pakistan

**Keywords:** Stroke, Eye movement disorders, Visual scanning exercises, Task specific approach

## Abstract

**Background:**

Impaired vision is one of the commonest and most disabling consequence following stroke. Among all visual impairments, eye movement disorders are found in 70% of stroke patients which include nystagmus, strabismus, gaze palsies, disconjugate eye movements and cranial nerve palsies. They have a wide ranging impact on balance and activities of daily livings by creating difficulties in maintaining normal alignment and appropriate movement of eyes. The purpose of this study was to examine the effects of visual scanning exercises in addition to task specific approach on balance and activities of daily livings in post stroke patients with eye movement disorders.

**Methods:**

This study is a randomized controlled trial and was conducted in the University of Lahore Teaching Hospital from May 2019 to October 2020. A sample of 64 patients was recruited and randomly allocated into experimental and control group. 32 patients in experimental group were treated with visual scanning exercises along with task specific approach and 32 patients in control group were treated with task specific approach alone. Pre and post assessment of balance and activities of daily livings was assessed on BERG BALANCE SCALE and BARTHEL INDEX SCALE at baseline and at 4^th^ week.

**Results:**

Intra-group analysis of BERG BALANCE SCALE in experimental group showed statistically significant result (*p* < 0.05) in all items except in items 4, 13 and 14 respectively. Intra-group analysis of BERG BALANCE SCALE in control group showed statistically significant result (*p* < 0.05) in items 3, 5, 8 and 12 respectively, whereas remaining all items showed statistically insignificant result. Intra-group analysis of BARTHEL INDEX SCALE in experimental group showed statistically significant result in all items (*p* < 0.05) except in items 9 and 10 respectively. Intra-group analysis of BARTHEL INDEX in control group showed statistically significant result (*p* < 0.05) in items 1, 3, 4 and 8 respectively whereas remaining all items showed statistically insignificant result. Inter-group analysis showed statistically significant result in total scores of BERG BALANCE SCALE (*p* = 0.000) and BARTHEL INEX SCALE (*p* = 0.033).

**Conclusion:**

Visual scanning exercises along with task specific approach were found to be more effective in comparison to task specific approach alone.

**Trial registration:**

Trial registration number: [IRCT20190717044237N1], trial registration date: 10/11/2019,

**Supplementary Information:**

The online version contains supplementary material available at 10.1186/s12883-022-02843-7.

## Background

Stroke is the second major cause of death after ischemic heart disease worldwide and the third major cause of death and disability [1]. Approximately, 6.6 million deaths are attributable to stroke worldwide with an estimated rate of overall prevalence exists 2.5% [2]. There is also a very high prevalence of stroke among Asians, increasing the overall burden of stroke up to 30–50% [3]. The annual projected incidence of stroke in Pakistan is about 250 per 100,000 population with estimated 350,000 new patients of stroke per year [4].

In addition to sensorimotor dysfunction, stroke may cause damage to primary visual cortex which interrupts the linkage to retinal receptor cells [5]. This leads to impaired vision that is one of the commonest and most disabling consequence following stroke and is reported in 60% of stroke population [6]. Among all visual impairments, eye movement disorders are found in 70% of stroke patients [7] which include nystagmus, strabismus, gaze palsies, disconjugate eye movements and cranial nerve palsies [8]. These eye movement disorders are caused due to damage of extraocular muscle of eye, damage to cranial nerves supplying to these eye muscles or to the neural pathways that are involved in controlling these nerves [7]. After stroke, patients with eye movement disorders present themselves clinically as, difficulty in maintaining an eye contact and to pay concentration on tasks, complaining of headaches and eye strain, closing or covering of one eye during the performance of different activities or during conversation because of diplopia or blurred vision, letters move about on page while reading, inability to see objects or person appearing abruptly from one side, constantly rubbing their eyes and squinting [9, 10]. Post stroke patients with these visual impairments may cause balance and gait deficits which leads to increased risk of fall [11]. Balance in stroke patients is maintained by the integration of visual, proprioceptive and vestibular feedback and sensory information coming from these systems influence motor control [12]. Vision play a crucial role in the control of balance. It can maintain bipedal upright stability during movement as a part of this combined sensory feedback system [13]. After stroke, eye movement disorders may impair brain’s ability to respond to these sensory information [14, 15]. As a result of this sensory conflict there is misalignment of body with special orientation that causes asymmetrical weight-bearing and these asymmetries result in balance impairments [16, 17]. In fact 16.7% to 83% of overall stroke survivors are reported to have balance problems [18]. Eye movement disorders can also have a wide ranging impact on activities of daily livings and independence by creating difficulties in maintaining normal alignment and appropriate movement of eyes. An immediate and precise analysis of visual symptoms is needed by the clinicians to establish and plan rehabilitation and treatment strategies for post stroke patients with eye movement disorders [8, 19, 20].

Different treatment modalities are used for the treatment of eye movement disorders such as, vision restoration therapy, eye movement training, neuro-eye therapy [21], and compensatory head posture exercises but still at present no standard therapeutic options are found because of lack of high-quality evidence-based research in this region [22]. According to the findings of Cochrane review [7], only two interventional studies have been investigated for the eye movement disorders. Both of these trials examined only the pharmacological interventions but not studied the rehabilitation outcomes. Authors of both studies concluded that there is inadequate research evidence to conclude the effects of therapeutic measures for the post stroke patients with eye movement disorders [23, 24].

It has been shown that visual scanning exercises, also known as compensatory mechanism of visual rehabilitation can improve visual scanning behavior among post stroke patients with eye movement disorders [25]. While, the task-specific approach is the standard treatment approach in which movement appears as an interface among various systems in the brain and is constructed around a goal and scrutinized by the environment [26].

Although, there are various treatment options present for eye movement disorders which are mentioned above but still there is a gap in literature regarding the best treatment option for eye movement disorder. Hence, the objective of this study was to determine the effects of visual scanning exercises in addition to task specific approach on balance and activities of daily livings in stroke patients with eye movement disorders.

## Methods

This study was a double blinded randomized controlled trial, conducted in the University of Lahore Teaching Hospital from May 2019 to October 2020. Data collection were started after taking permission from the Institutional Review Board, University of Lahore (IRB-UOL-FAHS/373-V/2018, dated: 20, Sep, 2018). Clinical trial was registered in Iranian registry of clinical trials on dated, 10/11/2019 and trial registration number was [IRCT20190717044237N1]. All methods were performed in accordance with the relevant guidelines and regulations. A sample size of 64 was calculated and inferred from Choi JU study by using following formula, [27] taking in consideration of 20% loss to follow-up.

$$\text{Sample size}=\frac{{{2\mathrm{SD}}^2({\mathrm Z}_{\alpha/2}+{\mathrm Z}_\beta)}^2}{\mathrm d^2}$$

Where, SD = Standard Deviation = 5.6, Z_a/2=_ type 1 error = 1.96, Z_β_ = at 80% power = 0.84, d = effect size = µ2-µ1 = 1.8.

**Fig. 1:**
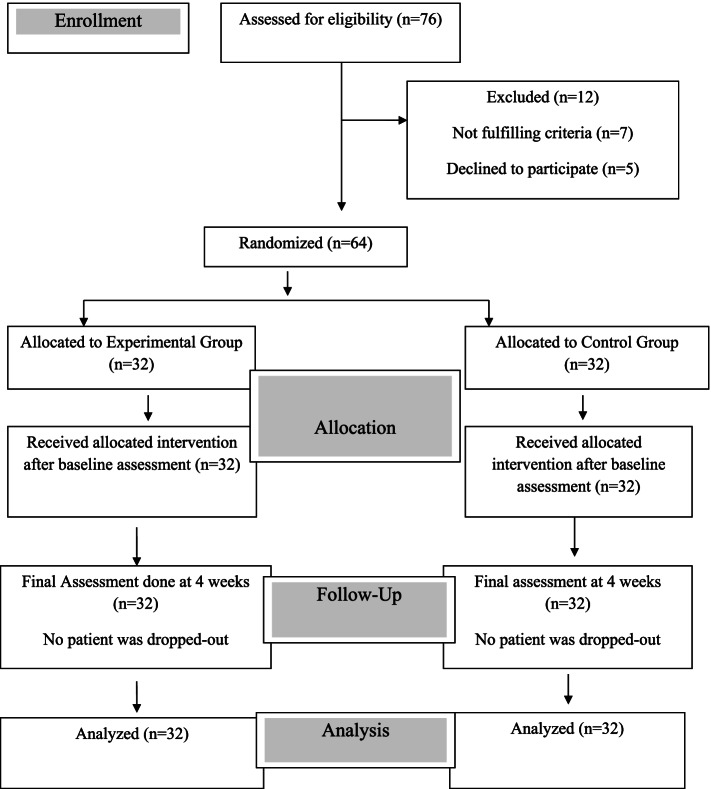
CONSORT 2010 Flow Sheet Diagram showing randomization and dropouts

Patients were recruited through non probability purposive sampling technique. All patients underwent detailed neurological examination. After that the patients who were having first stroke, age between 19–60 years, both genders, diagnosed with either ischemic or hemorrhagic stroke along with post stroke eye movement disorder by a neurophysician, patients in sub-acute phase 3–6 months, obtained a score of at least 25 and higher on mini mental state examination (MMSE), able to walk for a distance of at least 10 m with or without the help of an assistive device and were able to provide an informed consent were included in the study [28–30]. Patients who were having recurrent stroke, participated in other interventional or pharmacological studies, had any other organic disorder, orthopedic impairment or vestibular disorder with positive Dix hall pike test and demonstrate moderate to severe spasticity (a score of ≥ 2 on modified ashworth scale) in affected lower extremity, patients with visual field defects and patients with any other neurological disorder such as Parkinson’s disease, multiple sclerosis, and traumatic brain injury were excluded from the study [31–34]. Total 76 stroke patients were initially screened. From which 7 patients did not meet the inclusion criteria and 5 patients refused to take part in the study. Remaining 64 patients showed their willingness to participate in the study and were randomized into experimental and control groups. 32 patients in each group (Fig. 1) presenting CONSORT 2010 flow diagram for reporting randomized controlled trials [35]. The study was conducted according to the principles of the Declaration of Helsinki. Informed consent was taken from all the recruited patients.

### Randomization

This study had a double blinded randomized controlled design. Assessors and patients were blinded as they were not familiar about the group allocation and patients were not aware if they were performing visual scanning exercises or placebo exercises. All patients were allocated randomly to either experimental and control group by using computer generated random number table. All random numbers were kept in sealed envelopes. All envelops were kept by a third person who was not involved in this study. For each patient a sealed envelope was opened and mentioned group was allocated. Before the randomization process, all patients in experimental and control group were briefed about the study purpose and procedure and they were told that there will be no risks and harms to study patients.

### Data collection

Data were collected at baseline and at 4^th^ week i.e. at the completion of study by an independent assessor who was specialized in neurological physical therapy using two objective outcome measures. Balance was assessed by the berg balance scale and activities of daily livings were evaluated by the Barthel index scale.

### Berg Balance Scale (BBS)

The Berg Balance Scale (BBS) is an instrument used to assess the functional balance. It consist of 14 items related to balance, transfer, turning and stepping. Items are scored on a scale of 0–4 giving a total score of 56. It takes almost 15–20 min to complete [36].

### Barthel Index Scale (BI)

The Barthel Index (BI) is a scale to assess activities of daily livings (ADLs). It comprised of 10 items which include feeding, bathing, grooming, dressing, bowel, bladder, toilet use, transfer, mobility and stairs. Items were measured according to assistance and amount of time needed by the patient on a scale of 0 to 100. Where 0 indicates total dependence and 100 indicates complete independence [37]. According to guidelines by Shah et al. score between 0–20 showed “complete dependence”, 21–60 show severe dependence, 61–90 show moderate dependence and 91–99 show slight dependence [38].

### Interventions

After the baseline assessment patients in experimental group were treated with visual scanning exercises along with task-specific approach and patients in control group were treated with task specific approach along with placebo eye exercises. Patients in both groups were asked to performed tasks in five functional positions. The patients in experimental group were asked to perform eye movements (upward, downward, towards midline, laterally and diagonal movement of eyes) which were impaired in each functional position i.e. in supine lying, side lying to sitting, in sitting, during sit to stand, in standing and during walking. In each functional position patients were trained in two ways. First patients were asked to perform eye movements by naming the letters louder on flash cards which were displayed by the therapist on the affected side. Secondly, patients were asked to perform visual scanning exercises by naming the letters louder on a HART chart which was pasted on a wall. However the patients in control group were also instructed to perform different task specific exercises like feet balanced on mat and on gym ball in lying position while doing bridging. In sitting, patients were asked to perform sit to stand with and without assistance in front of table. Then patients were asked to practice standing with and without the help of an assistive device in front of table or against a wall, standing on one leg with other leg placed on balance ball and then they were trained walking on even and uneven surfaces or while holding a tray etc. Patients in both groups were given each exercise according to the guidelines mentioned in the previous study [39].

Experimental group was provided with task specific approach for 30 min and for another 15 min visual scanning exercises were given. Whereas, in control group task specific approach was given for 30 min and for another 15 min placebo eye exercises were given in which patients were asked to perform random eye movements in response to the torch light which was displayed by the therapist in different places of treatment room and patients were instructed to follow the light with their eyes.

**Fig. 2:**
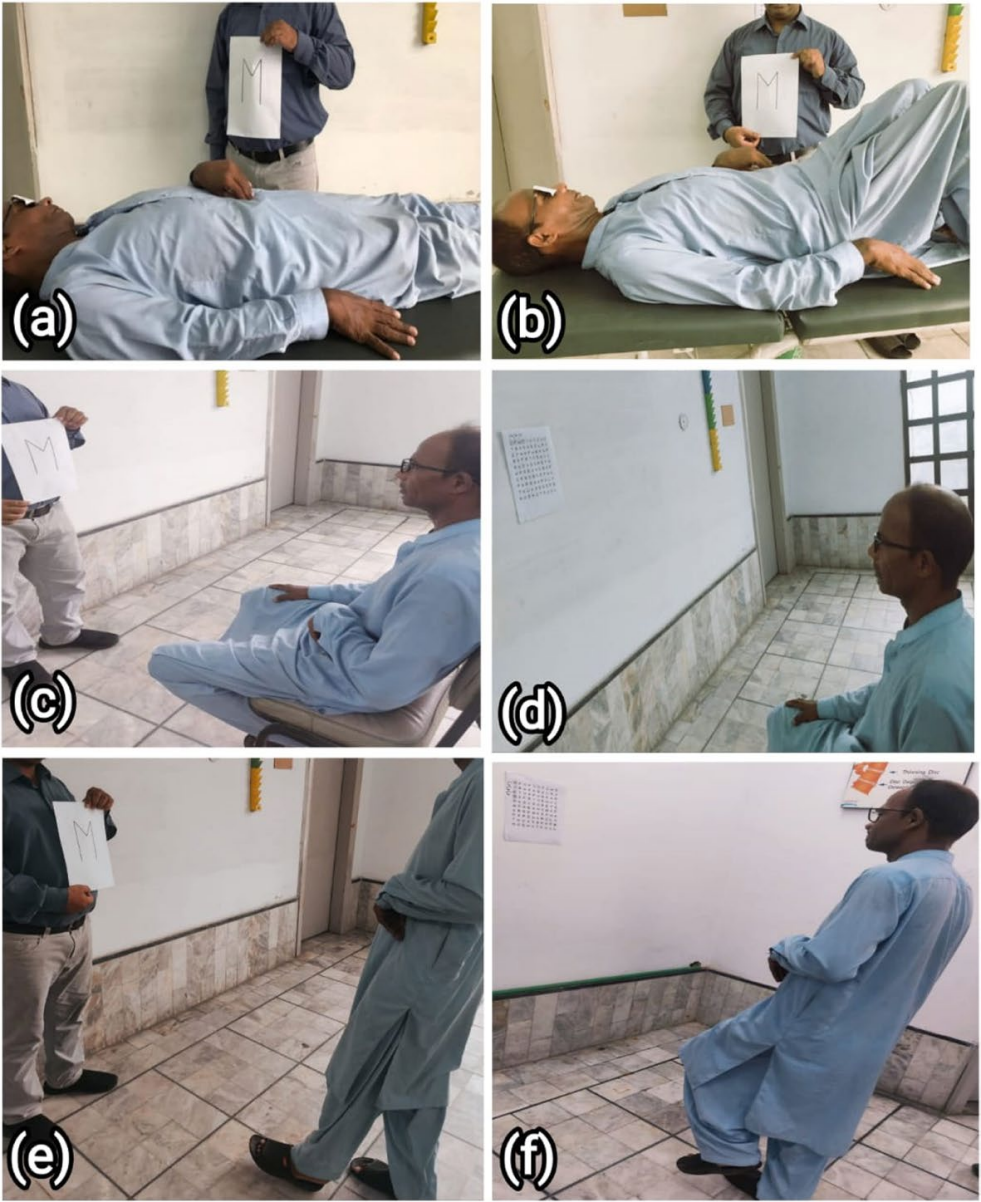
Performance of Visual Scanning Exercises in different Functional Positions

Both interventions were given 6 days per week, forty five minutes duration of each session for the period of one month. Total 24 sessions were performed. Intervention method that has been used in treating patients in experimental group is described in Fig. 2 showing performance of visual scanning exercises in different functional position. It has parts a, b, c, d, e and f. Detail of each part is given below.

- a = patient is performing visual scanning exercises in lying position while naming the letter showing on flash card by a physical therapist.
- b = patient is performing visual scanning exercises in bridging position while naming the letter showing on flash card by a physical therapist.
- c = patient is performing visual scanning exercises in sitting position while naming the letter showing on flash card by a physical therapist.
- d = patient is performing visual scanning exercises in sitting position while naming the letter on a HART chart placed on a wall.
- e = patient is performing visual scanning exercises in standing position while naming the letter showing on flash card by a physical therapist.
- f = patient is performing visual scanning exercises in standing on one leg while naming the letter on a HART chart placed on a wall.

### Statistical analysis

Data analysis were done using the SPSS version 21. Descriptive statistics were used to describe the demographic characteristics of study sample and were presented in the form of mean and standard deviation. The Shapiro–Wilk’s test was applied to test the normality of data and the result of test showed that the data was found normally distributed. A Parametric paired sample t-test was applied at the baseline and at 4^th^ week to find out the significance of changes within the groups (intra-group analysis). However, to find out the significance of changes between the groups (inter-group analysis) an independent t-test was applied. The level of significance of all measurements was set at *p* < 0.05.

## Results

### Clinical and demographic characteristics of the patients

There were 17 male and 15 female patients in experimental and 19 males and 13 females in control group. Ischemic stroke was found more prevalent in study participants, 20 patients in experimental and 18 patients in control group. However, the mean age of the study participants was found 55.63 ± 5.90 years in experimental and 54.38 ± 8.78 years in control group. There were more patients suffering with right hemiplegia 19 in experimental and 17 in control group. Most patients were having right dominant hand 24 in experimental and 25 in control group. 14 patients in experimental group and 12 patients in control group were using assisted device. The patients in both groups had different frequency of post stroke eye movement disorders like nystagmus, Strabismus, Saccadic eye movement, Smooth pursuit eye movement, Disconjugate eye movement and gaze palsies (Table 1).

**Table 1:** Clinical and demographic characteristics of the patients

Characteristics	Experiment Group Mean ± S.D	Control Group Mean ± S.D
Age (Years)	55.63 ± 5.90	54.38 ± 8.78
Gender		
Male	17	19
Female	15	13
Stroke type		
Ischemic stroke	20	18
Hemorrhagic stroke	12	14
Hemiplegic side		
Right	19	17
Left	13	15
Dominant hand		
Right	24	25
Left	8	07
Use of assistive device		
Yes	14	12
No	18	20
Post stroke eye movement disorders		
Nystagmus	6	5
Strabismus	5	5
Saccadic eye movement	6	5
Smooth pursuit eye movement	5	6
Disconjugate eye movement	5	5
Gaze palsies III, IV & VI	5	6

*SD* Standard Deviation

### Intragroup analysis

Intragroup analysis of BBS in experimental group showed statistically significant result (*p* < 0.05) in all items except in items 4, 13 and 14 respectively. However, intragroup analysis of BBS in control group showed statistically significant result (*p* < 0.05) in items 3, 5, 8 and 12 only. Intragroup analysis of BI scale in experimental group showed statistically significant result (*p* < 0.05) in all items except item 9 and 10, whereas in control group it showed statistically significant result (*p* < 0.05) in items 1, 3, 4 and 8 only. Intragroup analysis of total scores of BBS and BI showed statistically significant result (*p* = 0.000) in both experimental and control groups (Tables 2 and 3).

**Table 2:** Intragroup Comparison of BBS and BI Scale in Experimental Group

**S.No**	**Items of BBS and BI Scale**	**Pre-treatment Mean ± SD**	**Post-treatment Mean ± SD**	**Mean Difference**	***P*-Value**
1	BBS: Sitting to Standing	0.78 ± 0.42	1.38 ± 0.71	0.60 ± 0.29	0.001^*^
2	BBS: Standing unsupported	0.75 ± 0.44	1.22 ± 0.61	0.47 ± 0.17	0.004^*^
3	BBS: Sitting unsupported	0.91 ± 0.40	1.34 ± 0.60	0.43 ± 0.2	0.001^*^
4	BBS: Standing to sitting	0.81 ± 0.40	0.91 ± 0.47	0.1 ± 0.07	0.083
5	BBS: Transfers	0.72 ± 0.46	1.13 ± 0.61	0.41 ± 0.15	0.002^*^
6	BBS: Standing with eyes closed	0.75 ± 0.44	1.25 ± 0.67	0.50 ± 0.23	0.001^*^
7	BBS: Standing with feet together	0.72 ± 0.46	1.22 ± 0.75	0.50 ± 0.29	0.003^*^
8	BBS: Reaching forward with outstretched arm	0.88 ± 0.61	1.16 ± 0.63	0.28 ± 0.02	0.010^*^
9	BBS: Retrieving object from floor	0.81 ± 0.54	1.22 ± 0.55	0.41 ± 0.01	0.002^*^
10	BBS: Turning to look behind	0.78 ± 0.42	1.22 ± 0.71	0.44 ± 0.29	0.001^*^
11	BBS: Turning 360 degrees	0.72 ± 0.52	1.34 ± 0.87	0.62 ± 0.35	0.002^*^
12	BBS: Placing alternate foot on stool	0.78 ± 0.42	1.25 ± 0.76	0.47 ± 0.34	0.004^*^
13	BBS: Standing with one foot in front	0.69 ± 0.47	0.88 ± 0.66	0.19 ± 0.19	0.083
14	BBS: Standing on one foot	0.66 ± 0.48	0.84 ± 0.77	0.18 ± 0.29	0.136
BBS Total	Berg balance scale total Scores	10.75 ± 2.17	16.34 ± 2.88	5.59 ± 0.71	0.000^*^
1	BI: Feeding	2.19 ± 2.52	3.91 ± 3.75	1.72 ± 1.23	0.003^*^
2	BI: Bathing	1.72 ± 2.41	3.13 ± 2.46	1.41 ± 0.05	0.002^*^
3	BI: Grooming	1.56 ± 2.35	3.28 ± 2.41	1.72 ± 0.06	0.001^*^
4	BI: Dressing	2.03 ± 2.49	3.75 ± 3.59	1.72 ± 1.1	0.003^*^
5	BI: Bowels	1.88 ± 2.46	3.28 ± 3.50	1.40 ± 1.04	0.005^*^
6	BI: Bladder	2.03 ± 2.49	3.28 ± 3.50	1.25 ± 1.01	0.003^*^
7	BI: Toilet use	1.88 ± 2.46	3.28 ± 3.73	1.40 ± 1.27	0.010^*^
8	BI: Transfers	2.03 ± 2.49	3.91 ± 4.35	1.88 ± 1.86	0.003^*^
9	BI: Mobility	1.56 ± 2.35	2.50 ± 3.11	0.94 ± 0.76	0.083
10	BI: Stairs	1.41 ± 2.28	2.34 ± 3.11	0.93 ± 0.83	0.056
BI Total	Barthel Index Total Scores	18.28 ± 7.47	32.66 ± 12.69	14.38 ± 5.22	0.000^*^

*SD* Standard Deviation, *BBS* Berg balance scale, *BI* Barthel Index

**Table 3:** Intragroup Comparison of BBS and BI Scale in Control Group

S.No	Items of BBS and BI Scales	Pre-treatment Mean ± SD	Post-treatment Mean ± SD	Mean Difference	*P*-Value
1	BBS: Sitting to Standing	0.84 ± 0.45	0.94 ± 0.50	0.10 ± 0.05	0.184
2	BBS: Standing unsupported	0.78 ± 0.42	0.84 ± 0.45	0.06 ± 0.03	0.161
3	BBS: Sitting unsupported	0.94 ± 0.67	1.16 ± 0.81	0.22 ± 0.14	0.032^*^
4	BBS: Standing to sitting	0.84 ± 0.63	0.88 ± 0.66	0.04 ± 0.03	0.325
5	BBS: Transfers	0.75 ± 0.51	0.91 ± 0.59	0.16 ± 0.08	0.023^*^
6	BBS: Standing with eyes closed	0.78 ± 0.55	0.84 ± 0.63	0.06 ± 0.08	0.161
7	BBS: Standing with feet together	0.72 ± 0.46	0.75 ± 0.51	0.03 ± 0.05	0.325
8	BBS: Reaching forward with outstretched arm	0.91 ± 0.59	1.13 ± 0.61	0.22 ± 0.02	0.032^*^
9	BBS: Retrieving object from floor	0.84 ± 0.51	0.94 ± 0.56	0.1 ± 0.05	0.083
10	BBS: Turning to look behind	0.81 ± 0.47	0.88 ± 0.55	0.07 ± 0.08	0.161
11	BBS: Turning 360 degrees	0.75 ± 0.51	0.84 ± 0.68	0.09 ± 0.17	0.184
12	BBS: Placing alternate foot on stool	0.81 ± 0.40	0.94 ± 0.56	0.13 ± 0.16	0.044^*^
13	BBS: Standing with one foot in front	0.72 ± 0.46	0.84 ± 0.57	0.12 ± 0.11	0.103
14	BBS: Standing on one foot	0.69 ± 0.47	0.75 ± 0.51	0.06 ± 0.04	0.161
BBS Total	Berg balance scale total Scores	11.19 ± 2.18	12.63 ± 2.52	1.44 ± 0.34	0.000^*^
1	BI: Feeding	2.50 ± 2.54	3.13 ± 3.30	0.63 ± 0.76	0.044^*^
2	BI: Bathing	1.88 ± 2.46	2.19 ± 2.52	0.31 ± 0.06	0.161
3	BI: Grooming	1.88 ± 2.46	2.66 ± 2.54	0.78 ± 0.08	0.023^*^
4	BI: Dressing	2.19 ± 2.52	3.28 ± 3.27	1.09 ± 0.75	0.032^*^
5	BI: Bowels	2.19 ± 2.52	2.34 ± 2.54	0.15 ± 0.02	0.325
6	BI: Bladder	2.34 ± 2.54	2.81 ± 2.82	0.47 ± 0.28	0.083
7	BI: Toilet use	2.03 ± 2.49	2.50 ± 3.11	0.47 ± 0.62	0.184
8	BI: Transfers	2.19 ± 2.52	3.28 ± 3.73	1.09 ± 1.21	0.017^*^
9	BI: Mobility	1.56 ± 2.35	2.34 ± 3.59	0.78 ± 1.24	0.169
10	BI: Stairs	1.56 ± 2.35	1.72 ± 2.41	0.16 ± 0.06	0.662
BI total scores	Barthel Index total Scores	20.31 ± 7.72	26.25 ± 10.70	5.94 ± 2.98	0.000^*^

*SD* Standard Deviation, *BBS* Berg balance scale, *BI* Barthel Index

### Intergroup analysis

In Intergroup analysis before intervention no improvement was seen in BBS and BI scores (Table 4). After the period of 4 weeks, BBS showed statistically significant result (*p* < 0.05) in items 1, 2, 6, 7, 10 and 11 whereas, BI showed statistically insignificant result in all items. However, total scores of BBS (*p* = 0.000) and BI scale (*p* = 0.033) showed statistically significant result (Table 5).

**Table 4:** Intergroup Comparison of Experimental and Control Group for all items of BBS and BI Scale before intervention

S.No	Items of BBS and BI Scales	Experimental Group Mean ± SD	Control Group Mean ± SD	*P*-value (95% CI)
1	BBS: Sitting to Standing	0.78 ± 0.42	0.84 ± 0.45	0.567
2	BBS: Standing unsupported	0.75 ± 0.44	0.78 ± 0.42	0.772
3	BBS: Sitting unsupported	0.91 ± 0.39	0.94 ± 0.67	0.820
4	BBS: Standing to sitting	0.81 ± 0.40	0.84 ± 0.63	0.813
5	BBS: Transfers	0.72 ± o.46	0.75 ± 0.51	0.797
6	BBS: Standing with eyes closed	0.75 ± 0.44	0.78 ± 0.55	0.803
7	BBS: Standing with feet together	0.72 ± 0.46	0.72 ± 0.46	1.000
8	BBS: Reaching forward with outstretched arm	0.88 ± 0.61	0.91 ± 0.59	0.835
9	BBS: Retrieving object from floor	0.81 ± 0.54	0.84 ± 0.51	0.813
10	BBS: Turning to look behind	0.78 ± 0.42	0.81 ± 47	0.780
11	BBS: Turning 360 degrees	0.72 ± 0.52	0.75 ± 0.51	0.809
12	BBS: Placing alternate foot on stool	0.78 ± 0.42	0.81 ± 0.40	0.761
13	BBS: Standing with one foot in front	0.69 ± 0.47	0.72 ± 0.46	0.788
14	BBS: Standing on one foot	0.66 ± 0.48	0.69 ± 0.47	0.794
BBS Total	Berg balance scale total Scores	10.75 ± 2.17	11.19 ± 2.18	0.424
1	BI: Feeding	2.19 ± 2.52	2.50 ± 2.54	0.623
2	BI: Bathing	1.72 ± 2.41	1.88 ± 2.46	0.798
3	BI: Grooming	1.56 ± 2.35	1.88 ± 2.46	0.605
4	BI: Dressing	2.03 ± 2.49	2.19 ± 2.52	0.804
5	BI: Bowels	1.88 ± 2.46	2.19 ± 2.52	0.617
6	BI: Bladder	2.03 ± 2.49	2.34 ± 2.54	0.621
7	BI: Toilet use	1.88 ± 2.46	2.03 ± 2.49	0.802
8	BI: Transfers	2.03 ± 2.49	2.19 ± 2.52	0.804
9	BI: Mobility	1.56 ± 2.35	1.56 ± 2.35	1.000
10	BI: Stairs	1.41 ± 2.28	1.56 ± 2.35	0.788
BI Total	Barthel Index Total Scores	18.28 ± 7.47	20.31 ± 7.72	0.289

*SD* Standard Deviation, *BBS* Berg Balance Scale, *BI* Barthel Index

**Table 5:** Intergroup Comparison of Experimental and Control Group for all items of BBS and BI Scale after intervention

S.No	Items of BBS and BI Scales	Experimental Group Mean ± SD	Control Group Mean ± SD	*P*-value (95% CI)
1	BBS: Sitting to Standing	1.38 ± 0.71	0.94 ± 0.50	0.006^*^
2	BBS: Standing unsupported	1.22 ± 0.61	0.84 ± 0.45	0.007^*^
3	BBS: Sitting unsupported	1.34 ± 0.60	1.16 ± 0.81	0.296
4	BBS: Standing to sitting	0.91 ± 0.47	0.88 ± 0.66	0.827
5	BBS: Transfers	1.13 ± 0.61	0.91 ± 0.59	0.149
6	BBS: Standing with eyes closed	1.25 ± 0.67	0.84 ± 0.63	0.015^*^
7	BBS: Standing with feet together	1.22 ± 0.75	0.75 ± 0.51	0.005^*^
8	BBS: Reaching forward with outstretched arm	1.16 ± 0.63	1.13 ± 0.61	0.841
9	BBS: Retrieving object from floor	1.22 ± 0.55	0.94 ± 0.56	0.048
10	BBS: Turning to look behind	1.22 ± 0.71	0.88 ± 0.55	0.034^*^
11	BBS: Turning 360 degrees	1.34 ± 0.87	0.84 ± 68	0.012^*^
12	BBS: Placing alternate foot on stool	1.25 ± 0.76	0.94 ± 0.56	0.067
13	BBS: Standing with one foot in front	0.88 ± 0.66	0.84 ± 0.57	0.841
14	BBS: Standing on one foot	0.84 ± 0.77	0.75 ± 0.51	0.566
BBS Total	Berg balance scale total Scores	16.34 ± 2.88	12.63 ± 2.52	0.000^*^
1	BI: Feeding	3.91 ± 2.46	3.13 ± 3.30	0.380
2	BI: Bathing	3.13 ± 2.46	2.19 ± 2.52	0.137
3	BI: Grooming	3.28 ± 2.41	2.66 ± 2.54	0.316
4	BI: Dressing	3.75 ± 3.59	3.28 ± 3.27	0.587
5	BI: Bowels	3.28 ± 3.50	2.34 ± 2.54	0.225
6	BI: Bladder	3.28 ± 3.50	2.81 ± 2.82	0.558
7	BI: Toilet use	3.28 ± 3.73	2.50 ± 3.11	0.366
8	BI: Transfers	3.91 ± 4.35	3.28 ± 3.73	0.539
9	BI: Mobility	2.50 ± 3.11	2.34 ± 3.59	0.853
10	BI: Stairs	2.34 ± 3.11	1.72 ± 2.41	0.372
BI Total	Barthel Index Total Scores	32.66 ± 12.69	26.25 ± 10.70	0.033^*^

*SD* Standard Deviation, *BBS* Berg Balance Scale, *BI* Barthel Index

## Discussion

To the best of our knowledge, this is the first RCT to examine the effects of visual scanning exercises in addition to task-specific approach on balance and activities of daily livings in stroke patients with eye movement disorders. The purpose of visual scanning exercises was to encourage the subjects in learning in order to overcome their problems by increasing the accuracy and speed of eye movements on affected side [25]. 32 stroke patients in experimental group underwent visual scanning exercises along with task specific approach and 32 patients in control group were treated with task specific approach along with placebo eye exercises. At the end of 4 weeks of therapy patients in both groups brought about significant improvement in balance and activities of daily livings but experiment group showed more significant recovery in comparison to control group.

The findings of present study are similar to the study conducted by Arabzadeh S and co-workers [40]. In both studies, there was no statistical significant difference was seen at baseline in the BBS scores in both intragroup and intergroup analysis. However after the period of 4 weeks, significant improvement was seen in both studies in intra and intergroup analysis. The reason of significant improvement might be due to the average age of study participants. Mean age of study participants in both studies was less than 60 years. This lower age could be the reason of significant improvement. As it has been shown in literature that balance functions depend upon age and has been shown to diminish with age [18, 41]. Moreover, these findings may reflect the fact that activities performed in task specific approach were highly correlated with balance and daily living activities in stroke patients [42]. In another study Ahn et al. examined 30 patients which were randomly allocated to experimental and control group. In this study patients also showed significant improvement in BBS scores after the application of task-selective program [43]. In contrast to this, in another study conducted by Salbach et al. no improvement was observed in BBS scores of chronic stroke patients given a task specific walking exercises for the period of 6 weeks [44]. One more reason of significant recovery that was observed in present study might be that patients were in subacute phase 3–6 months post stroke and literature suggest that if patients receive rehabilitation care during this period they achieve maximum recovery in functional outcomes [45].

The findings of present study are also similar to the study conducted by Wyk AV and colleagues [39] who determined the combined effect of saccadic eye movement along with visual scanning exercises and task specific approach in post stroke patients with unilateral spatial neglect. The effect of these combined interventions after the period of four weeks showed statistically significant improvement (*p* = 0.004) in total scores of BI scale between group 1 and 2. This is similar to the findings of current study which also exhibited statistical significant improvement in total scores of BI scale as a result of inter-group analysis (*p* = 0.033) after the period of 4 weeks. In both studies, BI score improved to a larger extent in group 1 compared to group 2 showing that the level of dependence in group 1 decreased to a “moderate level of dependence” over the period of 4 weeks. Functional performance in ADLs also improved significantly in the participants of group 1 in both studies. It has been reported by the Nichols that balance functions are necessary for the successful performance of ADL functions and are associated with these activities [27]. In the present study balance functions improved significantly and as a result of these balance functions ADL functions also improved. However, patients’ performances in each single item of BI scale cannot be compared among two studies as scores of individual items performance were recorded in the present study only.

In contrast, a review article carried out by Pollock et al. [7] identified two studies. Both studies showed the pharmacological effect of interventions for nystagmus in only 5 stroke patients. Due to very limited number of stroke patients author was unable to draw any conclusion from these studies. Moreover, this review article did not find any RCT that examined the effect of interventions in stroke patients with eye movement disorder.

Likewise, another systematic review [19] included 11 studies of different types of visual impairments and examined their impact on quality of life of stroke patients but these studies did not include the study on eye movement disorders. Due to the absence of this particular visual impairment authors were unable to compare the effects of different visual impairments on quality of life of stroke patients. However, another systematic review included studies on eye movement which were missed in above systematic review along with 49 other studies on different visual impairments. Both systematic reviews highlighted the need for future research in this domain and also emphasized the need to conduct high quality randomized controlled trials to determine the effects of interventions in stroke patients with visual impairments [25].

Present study is a randomized controlled trial in which patients’ performance was recorded in all single items of BBS and BI scale besides calculating the mean difference in both groups. The observed mean difference in present study for the total scores of BBS and BI scale in experimental group was 5.59 ± 0.71 points and 14.38 ± 5.22 points respectively. And a noted mean difference in control group for the total scores of BBS and BI scale was 1.44 ± 0.34 points and 5.94 ± 2.98 points respectively. These values showed that more improvement has occurred in experimental group compared to control group. These findings are in line with a previous study which also showed a mean difference of more higher points for experimental group than the control group for both outcomes [27]. Hence, both studies demonstrate significant improvement in balance and ADLs. These findings may be explained by the fact that visual feedback therapies are proved to be effective in improving a symmetrical stance and in gaining a sitting balance [46, 47]. Moreover, task specific activities given to both groups also promote cerebral activation and brain reorganization and bring maximum improvement in functional performance [27

A retrospective study also reviewed the records of 220 patients suffering with either stroke or traumatic brain injury to investigate the frequency of eye movement dysfunctions and found that among 220 patients, cranial nerve palsy III and strabismus had the peak incidence rate [48]. However in the current study almost equal frequency of different eye movement disorders were found in both groups. Due to small sample size, frequency of each eye movement disorder was limited in each group. Due to this reason, we cannot evaluate that which particular type of eye movement disorder had highest rate and moreover which type of eye movement disorder had more impact on balance and activities of daily livings in stroke patients. Future studies should be carried out on large sample size to investigate this difference.

### Limitations

This study had few limitations regardless of its strengths. This study had recruited stroke patients with various types of eye movement disorders but owing to its small sample few numbers of patients were seen in each type, Due to this reason author was unable to conclude which type of eye movement disorder had more effect on study outcomes. Secondly, this study did not assess the effect of both therapies at follow-up so due to loss of follow-up long term effects of therapies were not investigated.

### Strengths

To the author’s knowledge this is the first RCT to assess the effects of visual scanning exercises in addition to task specific approach on balance and activities of daily livings in post stroke patients with eye movement disorders. Secondly, this study was a randomized trial so there may not have been selection bias. Thirdly, outcome measures tool used in this study take 15 to 20 min to administer so they can be used in clinical setting to assess balance and ADLs in stroke patients.

## Conclusion

From a clinical point of view this study suggested that visual scanning exercises along with task specific approach showed statistically significant improvement so it can be used to train balance and to improve activities of daily livings in stroke patients with eye movement disorders. In this study patient’s performance were recorded in all 14 items of BBS and 10 items of Barthel index scale instead of recording patient’s performance on sum of total scores only. This will help the clinicians to rule out the problem in specific item that needs improvement during rehabilitation. By just using the sum of total scores clinicians will not find the opportunity to localize the specific items of balance and daily livings activities that should be emphasized during stroke management.

## Supplementary Information

**Additional file 1.**

## Data Availability

All data generated or analyzed during this study are included in this published article [and its supplementary information files].

## Abbreviations

SD: Standard Deviation
MMSE: Mini mental status examination
BBS: Berg balance scale
BI: Barthel Index
ADLs: Activities of daily livings
SPSS: Statistical package for social sciences
RCT: Randomized controlled trial
CONSORT: Consolidated standards of reporting trials
