# Supplementary material for: Effects of visual scanning exercises in addition to task specific approach on balance and activities of daily livings in post stroke patients with eye movement disorders: a randomized controlled trial
Source: BMC Neurol. 2022 Aug 24;22:312. doi: 10.1186/s12883-022-02843-7 (PMC9400241; doi:10.1186/s12883-022-02843-7)
Supplement: Supplementary file 1 — Additional file 1. [file 12883_2022_2843_MOESM1_ESM.zip › Supplementary file 1 Berg balance scale.pdf]

BERG BALANCE TESTS AND RATING SCALE

Patient Name ____
Date ____
Location ____
Rater ____

ITEM DESCRIPTION SCORE (0-4) Sitting to standing _____ Standing unsupported _____ Sitting unsupported _____ Standing to sitting _____ Transfers _____ Standing with eyes closed _____ Standing with feet together _____ Reaching forward with outstretched arm _____ Retrieving object from floor _____ Turning to look behind _____ Turning 360 degrees _____ Placing alternate foot on stool _____ Standing with one foot in front _____ Standing on one foot _____ TOTAL _____

GENERAL INSTRUCTIONS

Please demonstrate each task and/or give instructions as written. When scoring, please record the lowest response category that applies for each item.

In most items, the subject is asked to maintain a given position for a specific time. Progressively more points are deducted if the time or distance requirements are not met, if the subject's performance warrants supervision, or if the subject touches an external support or receives assistance from the examiner. Subjects should understand that they must maintain their balance while attempting the tasks. The choices of which leg to stand on or how far to reach are left to the subject. Poor judgment will adversely influence the performance and the scoring.

Equipment required for testing are a stopwatch or watch with a second hand, and a ruler or other indicator of 2, 5 and 10 inches (5, 12 and 25 cm). Chairs used during testing should be of reasonable height. Either a step or a stool (of average step height) may be used for item #12.

1. SITTING TO STANDING

INSTRUCTIONS: Please stand up. Try not to use your hands for support.
( ) 4 able to stand without using hands and stabilize independently
( ) 3 able to stand independently using hands
( ) 2 able to stand using hands after several tries
( ) 1 needs minimal aid to stand or to stabilize
( ) 0 needs moderate or maximal assist to stand

2. STANDING UNSUPPORTED

INSTRUCTIONS: Please stand for two minutes without holding.
( ) 4 able to stand safely 2 minutes
( ) 3 able to stand 2 minutes with supervision
( ) 2 able to stand 30 seconds unsupported
( ) 1 needs several tries to stand 30 seconds unsupported
( ) 0 unable to stand 30 seconds unassisted

If a subject is able to stand 2 minutes unsupported, score full points for sitting unsupported. Proceed to item #4.

3. SITTING WITH BACK UNSUPPORTED BUT FEET SUPPORTED ON FLOOR OR ON A STOOL

INSTRUCTIONS: Please sit with arms folded for 2 minutes.
( ) 4 able to sit safely and securely 2 minutes
( ) 3 able to sit 2 minutes under supervision
( ) 2 able to sit 30 seconds
( ) 1 able to sit 10 seconds
( ) 0 unable to sit without support 10 seconds

4. STANDING TO SITTING

INSTRUCTIONS: Please sit down.
( ) 4 sits safely with minimal use of hands
( ) 3 controls descent by using hands
( ) 2 uses back of legs against chair to control descent
( ) 1 sits independently but has uncontrolled descent
( ) 0 needs assistance to sit

5. TRANSFERS

INSTRUCTIONS: Arrange chairs(s) for a pivot transfer. Ask subject to transfer one way toward a seat with armrests and one way toward a seat without armrests. You may use two chairs (one with and one without armrests) or a bed and a chair.
( ) 4 able to transfer safely with minor use of hands
( ) 3 able to transfer safely definite need of hands
( ) 2 able to transfer with verbal cueing and/or supervision
( ) 1 needs one person to assist
( ) 0 needs two people to assist or supervise to be safe

6. STANDING UNSUPPORTED WITH EYES CLOSED

INSTRUCTIONS: Please close your eyes and stand still for 10 seconds.
( ) 4 able to stand 10 seconds safely
( ) 3 able to stand 10 seconds with supervision
( ) 2 able to stand 3 seconds
( ) 1 unable to keep eyes closed 3 seconds but stays steady
( ) 0 needs help to keep from falling

7. STANDING UNSUPPORTED WITH FEET TOGETHER

INSTRUCTIONS: Place your feet together and stand without holding.
( ) 4 able to place feet together independently and stand 1 minute safely
( ) 3 able to place feet together independently and stand for 1 minute with supervision
( ) 2 able to place feet together independently but unable to hold for 30 seconds
( ) 1 needs help to attain position but able to stand 15 seconds with feet together
( ) 0 needs help to attain position and unable to hold for 15 seconds

8. REACHING FORWARD WITH OUTSTRETCHED ARM WHILE STANDING
INSTRUCTIONS: Lift arm to 90 degrees. Stretch out your fingers and reach forward as far as you can. (Examiner places a ruler at end of fingertips when arm is at 90 degrees. Fingers should not touch the ruler while reaching forward. The recorded measure is the distance forward that the finger reaches while the subject is in the most forward lean position. When possible, ask subject to use both arms when reaching to avoid rotation of the trunk.)
( ) 4 can reach forward confidently >25 cm (10 inches)
( ) 3 can reach forward >12 cm safely (5 inches)
( ) 2 can reach forward >5 cm safely (2 inches)
( ) 1 reaches forward but needs supervision
( ) 0 loses balance while trying/requires external support

9. PICK UP OBJECT FROM THE FLOOR FROM A STANDING POSITION
INSTRUCTIONS: Pick up the shoe/slipper which is placed in front of your feet.
( ) 4 able to pick up slipper safely and easily
( ) 3 able to pick up slipper but needs supervision
( ) 2 unable to pick up but reaches 2-5cm (1-2 inches) from slipper and keeps balance independently
( ) 1 unable to pick up and needs supervision while trying
( ) 0 unable to try/needs assist to keep from losing balance or falling

10. TURNING TO LOOK BEHIND OVER LEFT AND RIGHT SHOULDERS WHILE STANDING
INSTRUCTIONS: Turn to look directly behind you over toward left shoulder. Repeat to the right. Examiner may pick an object to look at directly behind the subject to encourage a better twist turn.
( ) 4 looks behind from both sides and weight shifts well
( ) 3 looks behind one side only other side shows less weight shift
( ) 2 turns sideways only but maintains balance
( ) 1 needs supervision when turning
( ) 0 needs assist to keep from losing balance or falling

11. TURN 360 DEGREES
INSTRUCTIONS: Turn completely around in a full circle. Pause. Then turn a full circle in the other direction.
( ) 4 able to turn 360 degrees safely in 4 seconds or less
( ) 3 able to turn 360 degrees safely one side only in 4 seconds or less
( ) 2 able to turn 360 degrees safely but slowly
( ) 1 needs close supervision or verbal cueing
( ) 0 needs assistance while turning

12. PLACING ALTERNATE FOOT ON STEP OR STOOL WHILE STANDING UNSUPPORTED
INSTRUCTIONS: Place each foot alternately on the step/stool. Continue until each foot has touched the step/stool four times.
( ) 4 able to stand independently and safely and complete 8 steps in 20 seconds
( ) 3 able to stand independently and complete 8 steps in >20 seconds
( ) 2 able to complete 4 steps without aid with supervision
( ) 1 able to complete >2 steps needs minimal assist
( ) 0 needs assistance to keep from falling/unable to try

13. STANDING UNSUPPORTED ONE FOOT IN FRONT
INSTRUCTIONS: (DEMONSTRATE TO SUBJECT) Place one foot directly in front of the other. If you feel that you cannot place your foot directly in front, try to step far enough ahead that the heel of your forward foot is ahead of the toes of the other foot. (To score 3 points, the length of the step should exceed the length of the other foot and the width of the stance should approximate the subject's normal stride width)
( ) 4 able to place foot tandem independently and hold 30 seconds
( ) 3 able to place foot ahead of other independently and hold 30 seconds
( ) 2 able to take small step independently and hold 30 seconds
( ) 1 needs help to step but can hold 15 seconds
( ) 0 loses balance while stepping or standing

14. STANDING ON ONE LEG
INSTRUCTIONS: Stand on one leg as long as you can without holding.
( ) 4 able to lift leg independently and hold >10 seconds
( ) 3 able to lift leg independently and hold 5-10 seconds
( ) 2 able to lift leg independently and hold = or >3 seconds
( ) 1 tries to lift leg unable to hold 3 seconds but remains standing independently
( ) 0 unable to try or needs assist to prevent fall

TOTAL SCORE (Maximum = 56: _______

***References**
Wood-Dauphinee S, Berg K, Bravo G, Williams JI: The Balance Scale: Responding to clinically meaningful changes. Canadian Journal of Rehabilitation, 10: 35-50,1997.

Berg K, Wood-Dauphinee S, Williams JI: The Balance Scale: Reliability assessment for elderly residents and patients with an acute stroke. Scand J Rehab Med, 27:27-36, 1995.

Berg K, Maki B, Williams JI, Holliday P, Wood-Dauphinee S: A comparison of clinical and laboratory measures of postural balance in an elderly population. Arch Phys Med Rehabil, 73: 1073-1083, 1992.

Berg K, Wood-Dauphinee S, Williams JI, Maki, B: Measuring balance in the elderly: Validation of an instrument. Can. J. Pub. Health, July/August supplement 2:S7-11, 1992.

Berg K, Wood-Dauphinee S, Williams JI, Gayton D: Measuring balance in the elderly: Preliminary development of an instrument. Physiotherapy Canada, 41:304-311, 1989.
